# Comparative Evaluation of Different Post Materials on Stress Distribution in Endodontically Treated Teeth Using the Finite Element Analysis Method: A Systematic Review

**DOI:** 10.7759/cureus.29753

**Published:** 2022-09-29

**Authors:** Vijetha Badami, Hemabhanu Ketineni, Sabiha PB, Sneha Akarapu, Satya Priya Mittapalli, Ayesha Khan

**Affiliations:** 1 Department of Conservative Dentistry and Endodontics, MNR Dental College and Hospital, Hyderabad, IND

**Keywords:** systematic review, fiber post, finite element analysis, stress distribution, post materials

## Abstract

The aim of this systematic review is to summarize and conclude findings to reveal the stress ranges developed by various post materials by finite element analysis. This, in turn, aids in the selection of better post material clinically. The electronic databases PubMed and Google Scholar were searched in this review by using specific inclusion and exclusion criteria. Among 14586 articles, 22 articles were included in this systematic review, as they satisfied the eligibility criteria. The search covered all articles published from 1997 to December 2021. All records identified were retrieved and imported into the Rayyan bibliographic software, which is a systematic review screening software. Later, data extraction and analysis of 22 articles were done. Twenty-two articles, which were all finite element analysis studies, were included. Among these, 18 studies used maxillary central incisor scanned models, two studies used a maxillary canine model, and the remaining two used the mandibular premolar model for finite element analysis. All these tooth models are restored with post models made of different materials. This systematic review revealed a difference in stress distribution in endodontically treated teeth when using different post materials. Among 22 studies, 15 studies evaluated glass fiber posts and the results showed that they induce less stress on restored endodontically treated teeth when compared to other posts, with maximum stress concentration at the cervical third of the root. Prefabricated posts like stainless steel and Titanium showed more stress on the restored tooth structure with stress concentration at the cervical and apical third of the root. Prefabricated zirconia also showed more stress on the restored tooth with maximum stress concentration at the middle third of the root.

## Introduction and background

An endodontically treated tooth is significantly weaker than a vital tooth and presents challenges in restoring it to form and function. Often, because of the minimal remaining tooth structure, posts are inserted to aid in the retention of the core. There are a number of options available in terms of materials for both the post and core, posts can be prefabricated or custom-made. The characteristics of the post include material elastic modulus, diameter, and height, which contribute greatly to the resistance to fracture of the restored tooth [1].

The selection of specific materials and techniques for the restoration of endodontically treated teeth is influenced by the changes that accompany root canal therapy, which include the amount of remaining tooth structure, physical changes in tooth structure, the anatomic position of the tooth, the occlusal forces on the tooth, the restorative requirements of the tooth, and the aesthetic requirements of the tooth [2].

Previously custom-cast posts and cores made of metals like gold, silver, palladium, and base metal alloys have been used. In recent times, non-metal posts like fiber-reinforced composites, glass fiber-reinforced posts, carbon fiber posts, and zirconia posts have been introduced [3]. Recently, they have been using PEEK (polyether ether ketone), which includes carbon fiber-reinforced (CFR)-PEEK, glass fiber reinforced (GFR)-PEEK, and PEKK (polyether ketone-ketone [1]. These metal-free posts have advantages like biocompatibility, corrosion resistance, and similar mechanical properties to a natural tooth, less expensive, less time-consuming, and in some situations less invasive than customized posts and cores [4]. The modulus of elasticity is directly proportional to stiffness. During transverse loading, stiffer posts undergo less deformation since they have high resistance to bending but they have a marked wedging effect, which increases the risk of fracture due to longitudinal loading [5].

Despite the availability of numerous* in-vitro* studies, it still remains undetermined as to which is the best post system, especially in terms of choice of materials. Some authors advise posts with a high modulus of elasticity and some recommend close to that of dentin and some say no significant difference between these two [4]. A lot of studies are published using finite element analysis (FEA) trying to show the stress distribution on the endodontically treated teeth by different post materials under defined conditions. Although intense research is conducted in this field, a consistent conclusion has not yet been established. The aim of this systematic review is to give an understanding of finite element analysis studies conducted in this field. This helps summarize and conclude findings to reveal the stress ranges developed by various post materials and in turn helps in best post material selection clinically.

## Review

Materials and methods

Study Design

This systematic review followed Preferred Reporting Items for Systematic Reviews and Meta-Analysis (PRISMA) guidelines. The use of checklists in the PRISMA is likely to improve the reporting quality of a systematic review and provides substantial transparency in the selection process of papers in a systematic review.

Focused Question

A PICOS question was formulated based on the population, intervention, comparison, outcomes, and study design. The PICOS question was which post material had developed high stresses and low stress in endodontically treated teeth when tested through finite element analysis. The population considered here were scanned models of extracted teeth, the intervention was a post while the comparison was done between different post materials, the outcome assessed was stress distribution on the models, and the study design considered was those including finite element analysis.

Literature Search

**Figure 1:**
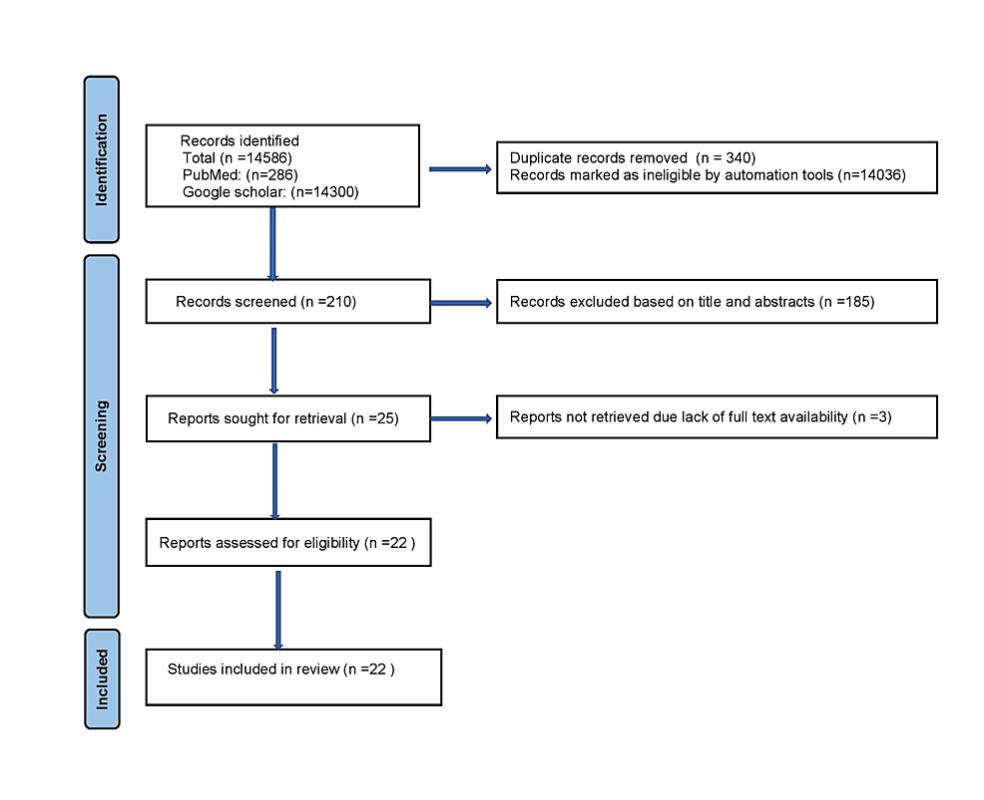
Flow Chart of the Search Strategy Used in This Systematic Review

An extensive search was carried out using electronic databases PubMed and Google Scholar along with a manual search to identify all articles related to stress distribution of different post and core materials using FEA. The electronic databases PubMed and Google Scholar were consulted by using the keywords “post and core materials, finite element analysis, stress distribution, and endodontically treated teeth.” The search included articles published in the English language from 1997 to December 2021. Identified articles were transferred into the Rayyan bibliographic software, which is systematic review screening software. The references of the selected articles were screened to identify other potentially relevant articles that have been missed during the initial search. Initial screening of identified articles was done by an assessment of title and abstract, and irrelevant articles were eliminated based on inclusion and exclusion criteria as mentioned below. The entire search process is depicted in Figure 1.

Inclusion and Exclusion Criteria

To be included, a study must be *in vitro*, evaluating stress distribution on human permanent teeth scanned models restored with different post materials using finite element analysis, and publications with full-text availability. Exclusion criteria included *in vivo* studies, reviews, studies not testing stress distribution by finite element analysis, studies using Bovine teeth models, and studies not including comparative evaluation of different post materials' stress distribution on restored teeth.

Data Extraction and Analysis

Two reviewers separately assessed all titles and abstracts based on inclusion and exclusion criteria. Initially, a combined assessment of 25 articles was done; later, the assessment of the articles was done separately. In case there was a difference of opinion, they would resolve it collectively. After that, data extraction and analysis of included articles were done. The data that were extracted included the journal, year of publishing, author, and tooth, which is tested, post materials, core materials, crown material, software of FEA, amount of force used, and area of its focus and results, as depicted in Tables 1-3.

**Table 1:** Data Extraction and Analysis of Included Studies in the Systematic Review

Journal and year	Author	Tooth model for testing	Models of post materials created for finite element analysis	Models of core materials created for finite element analysis	Models of crown materials created for finite element analysis	Software for FEA	Forces used and area of stress induction	Results
1. Biomaterials 2002	Pegoretti.A et al [6].	Maxillary central	Cast metal, carbon fiber, fiber reinforced, gold cast post	Composite core	Porcelain	MSC Marc (MSC software corporation)	100N, vertical 50N, oblique 10N, horizontal	Glass fiber composite shows less stress inside the root with force concentration in the cervical region.
2. Chinese Journal of Stomatology 2004	Chen.XT et al [7].	Maxillary central	Cast NI-CR (nickel-chromium), titanium alloy, cast gold, glass fiber, polyethylene fiber reinforced.	Composite core	PFM	Not mentioned	100N	Materials with elastic modulus similar to that of dentin such as polyethylene fiber-reinforced composite may be suitable for post restoration.
3. Operative Dentistry 2006	Barjau.E et al [8].	Maxillary central	Glass fiber, stainless steel			MSC Marc (MSC software corporation)	300N, palatal, 30 degrees	Glass fiber is better, the elastic modulus of the post is similar to that of dentin and the core has better performance.
4. Journal of Oral Rehabilitation 2006	Boschian Pest. L et al [9].	Lower first pre-molar	Carbon fiber, glass fiber, mineral	composite	All gold	MSC Marc(MSC software corporation)	486N, masticatory surface	Fiberglass reinforced composite, better than titanium/stainless steel.
5. European Journal of Dentistry 2007	Adanir.N et al [10].	Maxillary central incisors	Stainless steel, titanium, gold alloy, glass fiber, carbon fiber	Composite core		Structural Analysis Program 2000 (SAP 2000) (computer and structures, incorporated)	200N, vertical, 45 degrees, palatal	Glass fiber posts revealed more balanced stress distribution under functional forces.
6. Dental Material Journal 2008	Okada.DJ et al [11].	Maxillary central	Stainless steel, titanium, glass fiber post	Composite cores	Gold silver palladium alloy crown	MSC Marc (MSC Software Corporation)	Central occlusal surface, chewing force detected by sensors	Glass fiber post is more suitable for post fabrication.
7. Dental Material Journal 2009	Coelho.CS et al [12].	Maxillary central incisors	Cast Cu-Al (copper-aluminum) post, stainless steel, fiberglass, carbon fiber, zirconia dioxide, titanium	Composite core	Leucite-reinforced all ceramic	ANSYS (Analysis Systems) (ANSYS, Inc)	10N, palatal, 45 degrees	The use of custom cast posts, stainless steel posts, zirconia posts, and titanium posts resulted in increased stress in the post itself when compared to glass fiber posts and carbon fiber posts. A non-metallic post system results in improved mechanics of the weakened tooth.
8. Biomedical Materials 2010	Papadopoulos.T et al. [13].	Maxillary central incisors	Titanium, carbon fiber, glass fiber	Composite core	ceramic	MSC Marc (MSC Software Corporation)	400N, palatal, 45 degrees	Glass fiber post reduces the interfacial stress in post/core and post/tooth adhesion.

**Table 2:** Data Extraction and Analysis of Included Studies in the Systematic Review (Contd. 1)

Journal and year	Author	Tooth model for testing	Models of post materials created for finite element analysis	Models of core materials created for finite element analysis	Models of crown materials created for finite element analysis	Software for FEA	Forces used and area of stress induction	Results
9. Journal of Dental Research 2010	Santos.AF et al [14].	Pre-molar	Metallic post, fiber post	Resin core	All ceramic	MSC Marc(MSC software corporation)	300N, on the central ridge of the buccal cusp under 45 degrees	Fiber post generated low stress along the interface and increased stress in the root, post fracture less likely to occur in the root since its core and post failure indices were higher.
10. European Journal of Dentistry 2013	Shetty.PP et al [15].	Maxillary central incisors	Glass fiber post, zirconia	Composite core	Ceramic crown	MSC Marc (MSC Software Corporation)	100N, palatal, 45 degrees	The glass fiber post revealed homogenous stress distribution, in the zirconia post, the stress was concentrated in the post. Glass fiber can be used in well-conserved radicular tooth structure, whereas a zirconia post is used in weakened and grossly destructed teeth.
11. Journal of Peking University Health Sciences 2015	Zhou.TF et al [16].	Maxillary central incisors	CAD-CAM (Computer-Aided Design and Computer-Aided Manufacturing) zirconia, prefabricated zirconia, cast gold alloy post and core	Hot pressed porcelain core		Not mentioned	100N, vertical load, at 45 degrees palatal load	One piece zirconia post is more beneficial to disperse the bite force
12. Biomedical Material and Engineering 2015	Chen.A et al [17].	Maxillary canine	CAD-CAM zirconia, glass fiber, cast titanium, and cast gold	Same as post	Lithium disilicate glass-ceramic	ABAQUS (SIMULIA Structural solutions) (Dassault Systems)	100N, palatal, 45 degrees angle	CAD-CAM zirconia post system produced less stress when compared to CAD-CAM glass fiber, zirconia is the best post for badly damaged teeth followed by gold.
13. Journal of Indian Prosthetic Society 2016	Memon.S et al [18].	Maxillary central incisors	Glass fiber post and dentin post	Composite core	Porcelain crown	ABAQUS (SIMULIA Structural Solutions) (Dassault Systems)	100N, 135 degrees, palatal	Cervical region stress; stress in the cervical region was more for fiber posts.
14. Brazillian Dental Journal 2016	Diana.HH et al [19].	Maxillary canine	Glass fiber, carbon fiber, dentin post	Resin core	Metal ceramic	MSC Marc (MSC software corporation)	180N, lingual surface, 45 degrees	All three posts have the same stress distribution, high stress in the apical third of dentin; fiber and dentin posts exhibited similar stress values and distribution
15. Biomed Research International 2017	Lee KS et al [20].	Maxillary central incisors	Gold post and core, fiberglass, PEKK (Polyether ketone-ketone) post and core	Resin core	Ceramic crown	MSC Marc (MSC Software Corporation)	50N.palatal surface, 45 degrees	PEKK has a more favorable stress distribution than conventional post core materials but an increased probability of crown failure under long-term cyclic loading.
16. Journal of Dentistry 2018	Nokar.S et al [3].	Maxillary central incisors	Gold post and core, Ni-Cr post core, zirconia post and core, titanium, carbon fiber, glass fiber, quartz fiber, stainless steel, zirconia post	Composite core	Metal ceramic and all ceramic	ANSYS (Analysis Systems) (ANSYS, Inc)	100N, 45 degrees, palatal	Glass fiber produced less stress when compared to other posts

**Table 3:** Data Extraction and Analysis of Included Studies in the Systematic Review (Contd. 2)

Journal and year	Author	Tooth model for testing	Models of post materials created for finite element analysis	Models of core materials created for finite element analysis	Models of crown materials created for finite element analysis	Software for FEA	Forces used and area of stress induction	Results
17. Journal of Clinical and Experimental Dentistry 2019	De Andrade.GS et al [21].	Maxillary central incisors	CAD-CAM post and core nanoceramic, composite resin, hybrid ceramic, lithium disilicate, titanium, Y-TZP material (Yttria Stabilized Zirconia)	Similar to post	All ceramic	ANSYS (Analysis Systems) (ANSYS, Inc)	100N palatal, 45 degrees angle	Stress distribution on dentin was similar for all groups; these seem to be effective alternatives for conservative and aesthetic quality, crown core cement line stress is inversely proportional to the elastic modulus of the material. Post core cement line stress is directly related to the elastic modulus of the material.
18. Journal of Oral Biology and Craniofacial Research 2020	Nahar R et al [1].	Maxillary central incisors	FRC (Fiber-Reinforced Composite), CFR-PEEK (Carbon Fiber-Reinforced-Polyether Ether Ketone), GFR-PEEK(Glass Fiber Reinforced-Polyether Ether Ketone), PEKK (Polyether Ketone-Ketone)	Composite core	PFM and PEEK crown	ANSYS (Analysis Systems) (ANSYS, Inc)	100N, vertical force,45 degrees oblique force on the palatal surface	Both PFM (porcelain-fused metal crown) and PEEK with carbon fiber reinforced group observed that the post exhibited minimum von Mises stress, PEKK post maximum von Mises stress values.
19. JCD 2020	Tammineedi-s et al [22].	Maxillary central incisors	Dentin post, fiber post	Composite core	Porcelain crown	CATIA (computer-aided three-dimensional interactive application)(Dassault Systems)	100N, palatal surface, 45 degrees	Similar von Mises' stress value pattern of stress distribution; stress distribution is favorable in dentin posts.
20. Dental Research Journal 2021	Jafaris.et al [23].	Maxillary central incisors	NI-CR casting, glass fiber, titanium, zirconia post and core	Composite core	Zirconia monolithic crown	COMSOL Metaphysics software (Stockholm, Sweden)	100N, palatal, 135 degrees	Stress in the middle third of posts, glass fiber post stress distribution better than zirconia, cast post and core. Glass fiber post stress between the crown and cementoenamel junction if there is no ferrule more stress in the cervical region by glass fiber posts.
21. International Journal of Computerized Dentistry 2021	EIDR.et al [24].	Maxillary central incisors	CAD-CAM post made of FRC, high-density polymer, polymer-infiltrated ceramic network, metal alloy as control	Core same as post	Lithium disilicate crown	ANSYS (Analysis Systems) (ANSYS, Inc)		No statistical difference in all groups, no difference in unrestored fractures, and comparable resistance to cast metal post and core, so are acceptable alternatives.
22. Journal of the Indian Society of Pedodontics and Preventive Dentistry 2021	Patil DB.et al [25].	Maxillary central incisors	Carbon fiber, glass fiber, ever-stick	Composite	Porcelain crown	CATIA (computer-aided three-dimensional interactive application)(Dassault Systems)	200N, palatal surface, 45 degrees	Maximum stress was at the point of stress application, more stress was induced in this order: carbon fiber>glass fiber>ever-stick, more homogenous stress in ever-stick posts.

Results

The search of the electronic databases yielded 14586 articles; removing duplicates and unrelated articles resulted in a total of 210 articles. After reading their titles and abstracts, 185 studies were regarded as irrelevant or did not fulfill the inclusion criteria. Finally, the full texts of 22 articles that remained were studied in detail, as the full text of three articles full text was not available.

Among these, 18 studies pertained to the maxillary central incisor scanned model, two studies used the maxillary canine model, and the remaining two used the mandibular premolar model for finite element analysis. All these tooth models are restored with posts made of different materials. For the development of various post models, they used properties like modulus of elasticity and Poisson's ratio of these materials. In the case of prefabricated post models, they are restored with a composite core, and in the case of custom casting and computer-aided design and computer-aided manufacturing (CAD-CAM), the core is made of a similar material as the post. All these models are restored with crowns. To evaluate stress distribution in various different tooth models restored with different post materials force was generated on the model at a predetermined area by finite element analysis software and results were noted (Table 4).

**Table 4:** Results Table Showing Level of Stress Distribution and Areas of Stress Concentration of Different Post Materials

s.no	Post materials	No of studies evaluating the post material	High stress produced on models	Least stress produced on models	Areas of stress concentration on roots of models evaluated
1	Carbon fiber	6	2	-	Cervical third
2	Glass fiber	15	2	11	Cervical third
3	Ever-stick	1	-	1	Cervical third
4	CAD-CAM fiber-reinforced composite, high-density polymer, polymer-infiltrated ceramic network, nano ceramic, composite resin, hybrid ceramic, lithium disilicate, titanium, Y-TZP material, zirconia	4	-	1 (when compared with prefabricated)	-
5	CAD-CAM zirconia	3	-	2 (compared with prefabricated)	-
6	Titanium	7	5	-	Middle and apical third
7	Cast titanium	1	-	-	
8	Zirconia	6	4	-	Middle third
9	Cast zirconia	1	-	-	
10	Dentin post	3	-	2	Cervical third
11	Ni-Cr	6	2	2	Cervical and middle third
12	PEEK (Polyether Ether Ketone)	2	-	1	Coronal third
13	Stainless steel	5	4	-	Cervical and middle third

Discussion

The survival of endodontically treated and restored teeth depends on the amount of remaining coronal structure, restorative procedures, and material selection [26,27]. In particular, the preservation of at least one residual coronal wall or a circumferential 2-mm ferrule effect may contribute to overall tooth mechanical resistance [28]. Posts can be classified based on the elastic modulus, with metallic posts (prefabricated or cast metal posts), ceramic posts, and carbon fiber posts presenting high values and glass fiber posts presenting low elastic modulus [29]. The time needed for preparation, application, and esthetic performance have become important issues in daily practice, however, the strength and reliability of the system used are even more important [7]. During post and core treatment to restore a compromised endodontically treated tooth, the space is filled with a material that has a definite stiffness, unlike the pulp tissue and this creates an unnatural stress distribution within the tooth [1]. Posts that are stiff materials, unlike pulp tissue, create unnatural stresses on restored teeth [1]. Different post materials produce different stresses on restored teeth. FEA is the most widely used numerical method, allowing the reproduction of mechanical behavior under a mechanical load based on the properties of the materials [30]. To evaluate the stress distribution of these different post materials, many in vitro studies were conducted by using finite element analysis but a consistent conclusion has not been established. So in order to know which post material has better stress distribution in endodontically treated teeth, this systematic review was conducted.

After a detailed literature search, 22 articles were taken into consideration. Glass fiber posts have been used in 15 studies, of which they showed the least stress in 11 studies, prefabricated stainless steel used in five studies has been shown to produce more stresses on tooth structure in four studies, and prefabricated titanium used in seven studies showed high stresses in five studies. The modulus of elasticity (MOE) of enamel is around 80 GPa and that of dentin is 18.6GPa. Materials having MOE values close to enamel or dentin will have a better distribution of stress on restored teeth. The MOE value of glass fiber posts is around 40 Gpa, stainless steel, nickel-chromium (Ni-Cr), and zirconium are around 200, and that of titanium is 115 Gpa [1,11]. The forces generated to act on scanned models ranged from 100N-400N. A 100N force was frequently used in these studies, and the direction of these forces was mostly palatal. CAD-CAM posts made of various materials, such as fiber-reinforced composite (FRC), high-density polymer, polymer-infiltrated ceramic network, nanoceramic, composite resin, hybrid ceramic, lithium disilicate, titanium, Yttria-stabilized zirconia (Y-TZP) material, and zirconia, and tested in studies showed that there is no statistical difference in the stress distribution of these posts [21,24]. CAD-CAM/cast zirconia have better stress distribution than prefabricated zirconia [16]. Similarly, cast titanium has better stress distribution than prefabricated titanium. Cast metal post-and-core systems caused lower levels of stress compared to prefabricated metallic posts. When compared between carbon posts and fiber posts, the latter showed better stress distribution. Polyether ether ketone (PEEK) posts showed better stress distribution than polyether ketone-ketone (PEKK) posts [1]. Carbon fiber-reinforced-polyether ether ketone (CRF-PEEK), glass fiber reinforced-polyether ether ketone (GFR-PEEK), and PEKK materials can be used as materials of choice since they have similar stress distribution when compared to fiber-reinforced composite (FRC) post [1].

The stress distribution of glass fiber posts, even though homogenous, is mostly concentrated in the cervical region, whereas stainless steel, titanium, zirconia, and cast posts showed stress within the post, cervical, and apical regions of tooth structure. So fiber post fractures are less likely to occur in the root since its core and post failure indices were higher. Materials like fiber posts that show a homogenous stress distribution, have a modulus of elasticity similar or close to that of dentin. One study concluded that cast posts, stainless steel, titanium, and ceramic posts induced a more favorable stress distribution pattern in comparison with FRC posts [3].

The limitations of this systematic review are: all the post and core restorations were given crowns made of different materials, which could influence their stress distribution. The forces used in most of the studies are static forces, whereas in the oral cavity, there are masticatory forces that should be taken into consideration. Studies involving fiber posts are more when compared to other posts, which may have an influence on the outcome.

## Conclusions

Glass fiber posts induce a more homogeneous stress distribution with the least stresses on restored teeth as compared to all other posts. The maximum stress concentration is at the cervical third of the tooth. Prefabricated posts, in general, cause greater stress than custom posts. Prefabricated posts made of stainless steel and titanium exhibit more stresses on the restored tooth structure, with maximum stress concentration at the cervical and apical thirds of the root, whereas prefabricated zirconia posts manifest maximum stress concentration at the middle third.
